# Intraobserver Repeatability and Interobserver Reproducibility of Foveal Cone Density Measurements in *CNGA3-* and *CNGB3*-Associated Achromatopsia

**DOI:** 10.1167/tvst.9.7.37

**Published:** 2020-06-26

**Authors:** Michalis Georgiou, Katie M. Litts, Navjit Singh, Thomas Kane, Emily J. Patterson, Nashila Hirji, Angelos Kalitzeos, Alfredo Dubra, Michel Michaelides, Joseph Carroll

**Affiliations:** 1 UCL Institute of Ophthalmology, University College London, London, UK; 2 Moorfields Eye Hospital NHS Foundation Trust, London, UK; 3 Department of Ophthalmology & Visual Sciences, Medical College of Wisconsin, Milwaukee, WI, USA; 4 Department of Ophthalmology, Stanford University, Palo Alto, CA, USA

**Keywords:** adaptive optics, inherited retinal diseases, achromatopsia, cone photoreceptors, repeatability, reproducibility

## Abstract

**Purpose:**

To examine repeatability and reproducibility of foveal cone density measurements in patients with *CNGA3-* and *CNGB3*-associated achromatopsia (ACHM) using split-detection adaptive optics scanning light ophthalmoscopy (AOSLO).

**Methods:**

Thirty foveae from molecularly confirmed subjects with ACHM, half of whom harbored disease-causing variants in *CNGA3* and half in *CNGB3,* underwent nonconfocal split-detection AOSLO imaging. Cone photoreceptors within the manually delineated rod-free zone were manually identified twice by two independent observers. The coordinates of the marked cones were used for quantifying foveal cone density. Cone density and difference maps were generated to compare cone topography between trials.

**Results:**

We observed excellent intraobserver repeatability in foveal cone density estimates, with intraclass correlation coefficients (ICCs) ranging from 0.963 to 0.991 for *CNGA3* and *CNGB3* subjects. Interobserver reproducibility was also excellent for both *CNGA3* (ICC = 0.952; 95% confidence interval [CI], 0.903–1.0) and *CNGB3* (ICC = 0.968; 95% CI, 0.935–1.0). However, Bland-Altman analysis revealed bias between observers.

**Conclusions:**

Foveal cone density can be measured using the described method with good repeatability and reproducibility both for *CNGA3*- and *CNGB3*-associated ACHM. Any degree of bias observed among the observers is of uncertain clinical significance but should be evaluated on a study-specific basis.

**Translational Relevance:**

This approach could be used to explore disease natural history, as well as to facilitate stratification of patients and monitor efficacy of interventions for ongoing and upcoming ACHM gene therapy trials.

## Introduction

Achromatopsia (ACHM) presents clinically from birth or early infancy, with poor visual acuity, nystagmus, photophobia, color vision loss in all three axes, and substantially reduced or absent cone photoreceptor function. Genetically, disease-causing variants have been reported in *CNGA3* (ACHM2, OMIM600053),^1^^,^^2^ *CNGB3* (ACHM3, OMIM605080),^3^ *GNAT2* (ACHM4, OMIM139340)*,*^4^^,^^5^ *ATF6* (ACHM7, OMIM616517)*,*^6^^,^^7^ *PDE6H* (ACHM6,OMIMI610024),^8^ and *PDE6C* (ACHM5, OMIM600827).^9^^,^^10^ Variants in *CNGA3* and *CNGB3* are responsible for 70% of the reported cases^11^ and are the most well-studied genotypes.^12^^–^^15^ With ongoing gene therapy trials, there is a pressing need to develop reliable and repeatable outcome metrics.^16^ Given that ACHM affects the cone photoreceptors, such metrics should logically focus on assessing cone function and/or structure.

Adaptive optics scanning light ophthalmoscopy (AOSLO) allows transverse cellular resolution of the cone mosaic in vivo and has been used for in-depth phenotyping in a range of inherited retinal diseases.^17^^–^^20^ Early investigations with confocal reflectance AOSLO identified “dark spaces” in the ACHM cone mosaic, as well as increased cone spacing and/or decreased cone density.^21^^–^^24^ Marked variability in the cone mosaic has been observed across patients with ACHM.^23^^,^^25^^–^^28^ Nonconfocal split-detection AOSLO demonstrated that inner segment structure remained in these “dark spaces.” The degree of remnant inner segment structure may be important for participant selection for gene therapy trials and could be used to identify patients most likely to benefit from cone-directed rescue. Peak foveal cone density is a widely used metric, both as an anchor for locating other regions of interest and as a statistic in its own right; however, there have been relatively few studies examining the repeatability of these measurements.

Tanna et al.^29^ examined cone density measurements in patients with Stargardt disease and *RPGR*-associated retinopathy and showed that, for both conditions, measurements using split-detection AOSLO images were more reliable and repeatable than measurements using confocal AOSLO images. However, the reliability and repeatability differed between the two pathologies, suggesting that the degree and pattern of remnant cone structure may affect the measurements, and thus reliability could be disease dependent. Abozaid et al.^30^ examined reliability and repeatability of manual cone density measurements in a pilot study of seven subjects with ACHM (five with *CNGB3*-ACHM and two with *CNGA3*-ACHM) and found a strong observer effect owing to varying degrees of observer experience. Langlo et al.^13^ looked at repeatability of a single observer in evaluating the peak foveal cone density in *CNGB3*-ACHM and reported excellent intraclass correlation coefficient (ICC). However, no study has evaluated the repeatability of foveal cone density measurements in *CNGA3*-ACHM or interobserver reproducibility of foveal cone density measurements in ACHM.

Here we examine the intraobserver repeatability and interobserver reproducibility of foveal cone density measurements, both for *CNGA3*- and *CNGB3*-ACHM, in 15 foveae from each genotype. These data provide important baseline information for subsequent studies of the foveal cone mosaic in ongoing and upcoming ACHM gene therapy trials.

## Methods and Materials

### Subjects

The Ethics Committees of Moorfields Eye Hospital and the Medical College of Wisconsin approved the study. Written informed consent was obtained from all subjects after explanation of the nature and possible consequences of the study. The research followed the tenets of the Declaration of Helsinki. Subjects with likely disease-causing sequence variants in *CNGA3* or *CNGB3* were recruited from Moorfields Eye Hospital (London, UK) and the Medical College of Wisconsin, Milwaukee.

### AOSLO Imaging of the Photoreceptor Mosaic

#### Image Acquisition

All subjects were imaged using one of two similar AOSLO systems, previously described,^31^ housed at either Moorfields Eye Hospital or the Medical College of Wisconsin. Pupil dilation and cycloplegia were achieved by instilling one drop of phenylephrine hydrochloride (2.5%) and tropicamide (1%) in each eye prior to imaging. Confocal and split-detection AOSLO images of the photoreceptor mosaic were obtained across the foveal region. The imaging light source was a 790-nm super-luminescent diode (Superlum, Carrigtohill, Cork, Ireland). Image sequences were recorded as AVI files, of 150 to 200 frames, at 1°, 1.5°, and/or 1.75° fields of view. A desinusoiding algorithm was applied to each image sequence, and individual frames were selected,^32^ registered,^33^ and averaged to increase signal-to-noise ratio for subsequent analysis. The final images were combined into a single montage (Adobe Photoshop; Adobe Systems, Inc., San Jose, CA, USA) either manually or semiautomattically.^34^ Where possible, images taken using 1° field of view were analyzed due to their higher resolution.

#### Fovea Selection

A total of 32 subjects with *CNGA3-*ACHM and 53 subjects with *CNGB3*-ACHM were recruited and imaged for this study. Of these subjects, 15 and 30 had analyzable foveae, respectively. All 15 *CNGA3* foveae were included for analysis. Fifteen foveae with *CNGB3*-ACHM were selected by a third experienced observer (EJP) to be representative of the range of variability of the disease. All foveae were selected from different subjects.

#### Image Scaling

The linear scale of the AOSLO images for each subject ($S_{R(x)}^{'}$; units: µm/pixel) was estimated using the following equation:

(1) $$\begin{array}{c} S_{R\left(x\right)}^{'}=\frac{T}{f_{l}T_{s}}\left(\frac{180}{\pi }\right)RMF\left(\frac{l_{A}}{l_{A,0}}\right) \end{array}$$

where *T* represents the periodicity of a Ronchi ruling (µm/cycles), *f_l_* represents the focal length of the model eye in our system (µm), *T_s_* represents the sampling period between lines in the Ronchi ruling (pixels/cycle), *RMF* represents the assumed retinal magnification factor (291 µm/degree) of an eye with a 24.0-mm axial length (represented by *l*_*A*,0_), and *l_A_* represents the axial length of the subject's eye in millimeters (measured with an IOL Master; Carl Zeiss Meditec, Inc., Jena, Germany).

#### Foveal Cone Density Measurements

The rod-free zone, an area devoid of rods that typically appears hyporeflective on confocal AOSLO in ACHM, was delineated manually (Adobe Photoshop; Adobe Systems, Inc.) in all foveae by a single observer (MG) using the AOSLO montage. For each fovea (15 *CNGA3* and 15 *CNGB3*), cones within the delineated rod-free zone were manually identified twice by two observers (MG and KML), both experienced with AOSLO image analysis in ACHM. Each trial was separated by at least 1 week. The observers were masked to their previous cone identifications, as well as those of the other observer. The cone coordinate arrays were extracted (Mosaic Analytics; Translational Imaging Innovations, Inc., Hickory, NC, USA) and used to assess the foveal cone density, which was calculated for each trial of both observers. To determine foveal cone density (cones/mm^2^), a 55-µm × 55-µm sliding window was used to assess the cone density at each cone coordinate within the coordinate array using custom MATLAB software (MathWorks, Inc., Natick, MA USA). The cone coordinate location with the greatest value was identified as the location of maximum cone density for the area analyzed. The location of the maximum foveal cone density was recorded for both trials for each observer to examine the displacement.

For each trial, cone density at each cone coordinate within the foveal area counted was mapped. A difference map was created by calculating the absolute values of the difference in cone density at each overlapping pixel between the trials of an observer. The locations of maximum foveal cone density were plotted on the difference maps for comparison between the trials for each observer. For each fovea, this displacement was calculated in pixels (maximum foveal cone density location from trial 1 minus maximum foveal cone density location from trial 2) and converted to microns using the µm/pixel scale of the image.

### Statistical Methods

Statistical analysis was performed with IBM SPSS Statistics for Windows (version 22.0; SPSS, Inc., Armonk, NY, USA). The bias, limits of agreement (LOAs), and 95% confidence intervals (CIs) for the bias and LOA were calculated following the methods of Bland and Altman.^35^^,^^36^ For all data sets, normality was assessed using the Shapiro-Wilk normality test. Where normality could not be confirmed, nonparametric tests were used. The specific tests used are included alongside each result, as appropriate. ICCs were calculated for raw or log-transformed measurements as appropriate using R and the ICC package (version 2.3.0).

## Results

### Subject Demographics

The mean (SD; range) age for *CNGA3* and *CNGB3* subjects was 28.7 (12.6; 14–64) years and 24.6 (9.3; 15–51) years, respectively. There was no difference in the distribution of age in the two genotypes (Mann-Whitney, *z* = 0.788, *P* = 0.430). Subject demographics and genetics are summarized in the Supplementary Table.

### Intraobserver Repeatability

The mean test-retest difference was calculated from the absolute value of the differences between foveal cone density measurements for each observer, for each genotype separately. The mean test-retest difference between the two trials was between 1496 and 2466 cones/mm^2^, although there was significant variability in the individual differences of the 60 pairs of foveal cone density measurements, ranging from as low as 124 cones/mm^2^ to as high as 7763 cones/mm^2^. For observer 1, the mean (SD; range) absolute difference for *CNGA3* and *CNGB3* was 1496 (1211; 433–5476) cones/mm^2^ and 1576 (1154; 256–4675) cones/mm^2^, respectively. For observer 2, the mean (SD; range) absolute difference for *CNGA3* and *CNGB3* was 1647 (1873; 151–7763) cones/mm^2^ and 2466 (2319; 124–7674) cones/mm^2^, respectively.

**Figure 1.**
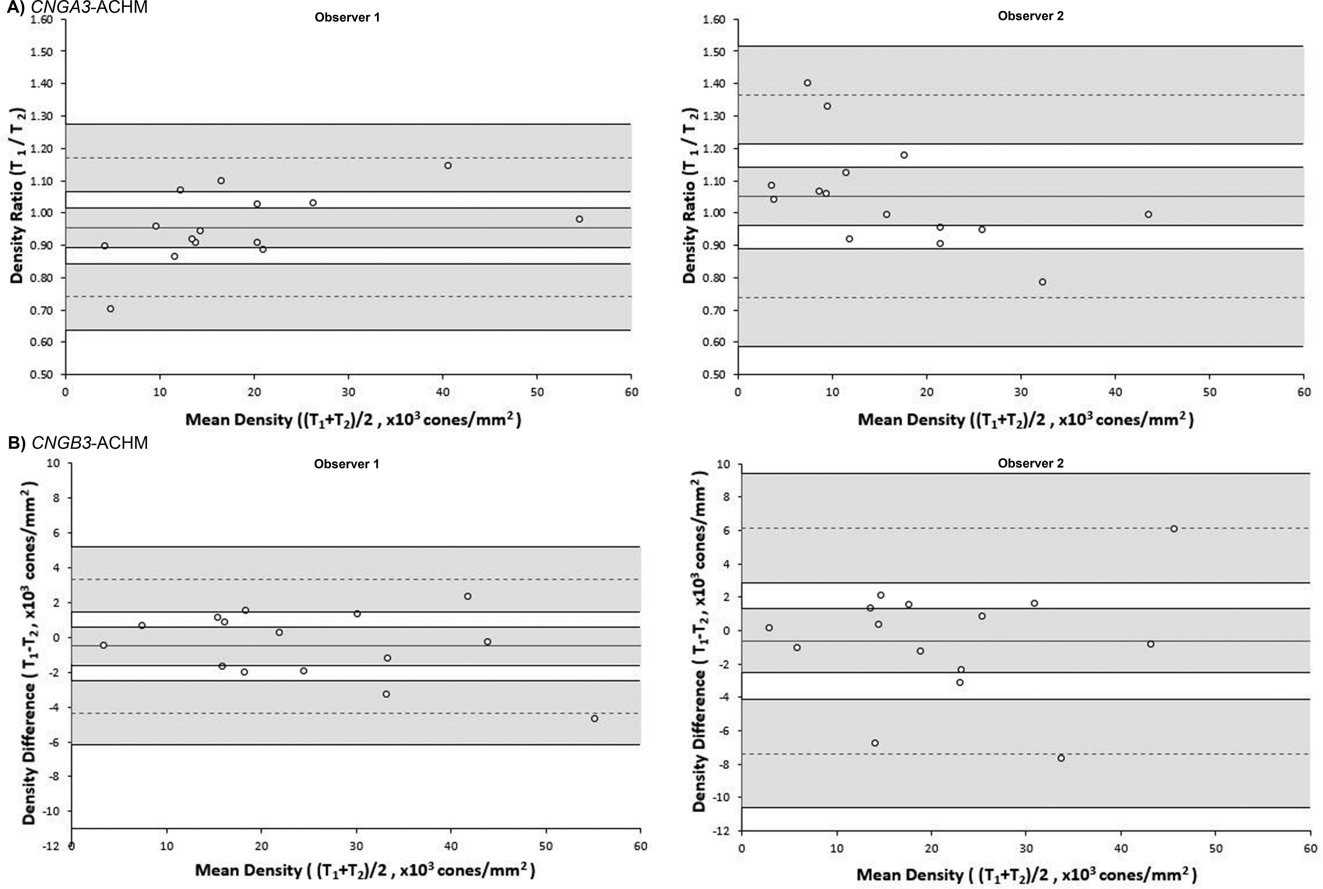
Bland-Altman plots illustrating the intraobserver repeatability of foveal cone density for (A) *CNGA3*-ACHM and (B) *CNGB3*-ACHM for observers 1 and 2. The mean foveal cone density difference (bias) is represented by the *central solid black line*, while the *dashed lines* represent the limits of agreement for the bias. *Shaded regions* represent the confidence intervals for the bias and limits of agreement. The data for *CNGA3*-ACHM were not normally distributed, so the ratio was used instead of the difference. T, trial.

Excellent intraobserver repeatability was observed for both *CNGA3* and *CNGB3* measurements of foveal cone density, for both observers, as shown by the ICC values provided in Table 1 and the Bland-Altman plots in Figure 1. The distribution of differences appears homoscedastic as a function of the mean, without proportional bias for either genotype or observer.

**Table 1.** Results and Intraobserver Repeatability of Peak Cone Density Measurements

	*CNGA3*		*CNGB3*	
Metric	Observer 1	Observer 2	Observer 1	Observer 2
Median; IQR (cones/mm^2^)	14,380; 9358	11,878; 12,778	21,917; 17,447	18,951; 16,799
Coefficient of variation (%)	9.33^a^	11.08^a^	5.47	10.97
ICC (95% CI)	0.983 (0.966 to 1.0)	0.979 (0.957 to 1.0)	0.991 (0.981 to 1.0)	0.963 (0.926 to 1.0)
Bland-Altman analysis^b^				
Bias (95% CI)	−4.48% (+1.58% to −10.54%)	+5.18% (−3.69% to +14.05%)	−508 cones/mm^2^ (−1589 to +574)	−605 cones/mm^2^ (−2514 to +1304)
Upper LOA (95% CI)	+16.95% (+6.60% to +27.31%)	+36.56% (+21.40% to +51.72%)	+3320 cones/mm^2^ (+1470 to +5169)	+6152 cones/mm^2^ (+2887 to +9417)
Lower LOA (95% CI)	−25.92% (−15.56% to −36.28%)	−26.20% (−11.04% to −41.36%)	−4335 cones/mm^2^ (−2485 to −6184)	−7362 cones/mm^2^ (−4097 to −10,627)

IQR, interquartile range.

a Logarithmic transformation was applied and coefficient of variation is computed using the antilog of the within-subject standard deviation.

b *CNGA3* measurements were nonnormally distributed; therefore, we report percentages and Bland-Altman analysis after logarithmic transformation.

### Interobserver Reproducibility

To assess interobserver reproducibility, we took the mean foveal cone density from the two trials within each observer. The data were analyzed individually for each genotype after logarithmic transformation due to nonnormal distribution. The mean (SD; range) absolute difference in foveal cone density between the two observers was 2861 (3004; 384–10,915) cones/mm^2^ and 3561 (3284; 563–10,187) cones/mm^2^ for *CNGA3* and *CNGB3*, respectively. Table 2 summarizes the interobserver reproducibility metrics.

**Table 2.** Interobserver Reproducibility of Peak Cone Density Measurements

Metric	*CNGA3*	*CNGB3*
Coefficient of variation (%)^a^	16.71	13.88
ICC (95% CI)	0.952 (0.903 to 1.0)	0.968 (0.935 to 1.0)
Bland-Altman analysis		
Bias (95% CI)	+19.21% (+8.95% to +29.47%)	+16.99% (+9.96% to +24.01%)
Upper LOA (95% CI)	+55.53% (+37.98% to +73.07%)	+41.83% (+29.83% to +53.84%)
Lower LOA (95% CI)	−17.11% (+0.44% to −34.66%)	−7.86% (+4.14% to −19.87%)

a Logarithmic transformation was applied and coefficient of variation is computed using the antilog of the within-subject standard deviation.

**Figure 2.**
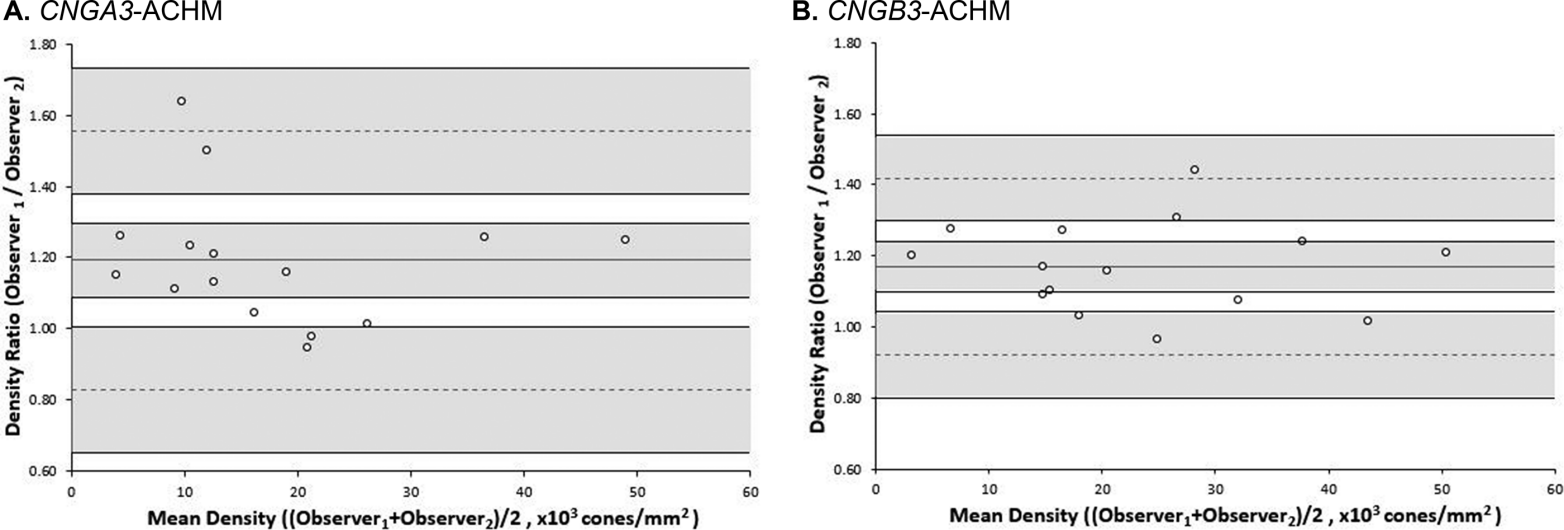
Bland-Altman plots illustrating the interobserver reproducibility of foveal cone density for (A) *CNGA3*-ACHM and (B) *CNGB3*-associated ACHM. The mean foveal cone density difference (bias) is represented by the *central solid black line*, while the *dashed lines* represent the 95% limits of agreement for the bias. *Shaded regions* represent the confidence intervals for the bias and limits of agreement. The data were not normally distributed, so the ratio was used instead of the difference.

There was a high ICC between the two observers for *CNGA3*-ACHM (ICC, 0.952; 95% CI, 0.903–1.0) and *CNGB3*-ACHM (ICC, 0.968; 95% CI, 0.935–1.0). In subsequent Bland-Altman analysis (Fig. 2), the distribution of differences was homoscedastic as a function of the mean. In contrast to intraobserver analysis, proportional bias was observed in Bland-Altman plots for both genotypes of similar degree (*CNGA3*, 19.21%; *CNGB3*, 16.99%). Higher values were associated with observer 1 and lower with observer 2 for both genotypes.

**Figure 3.**
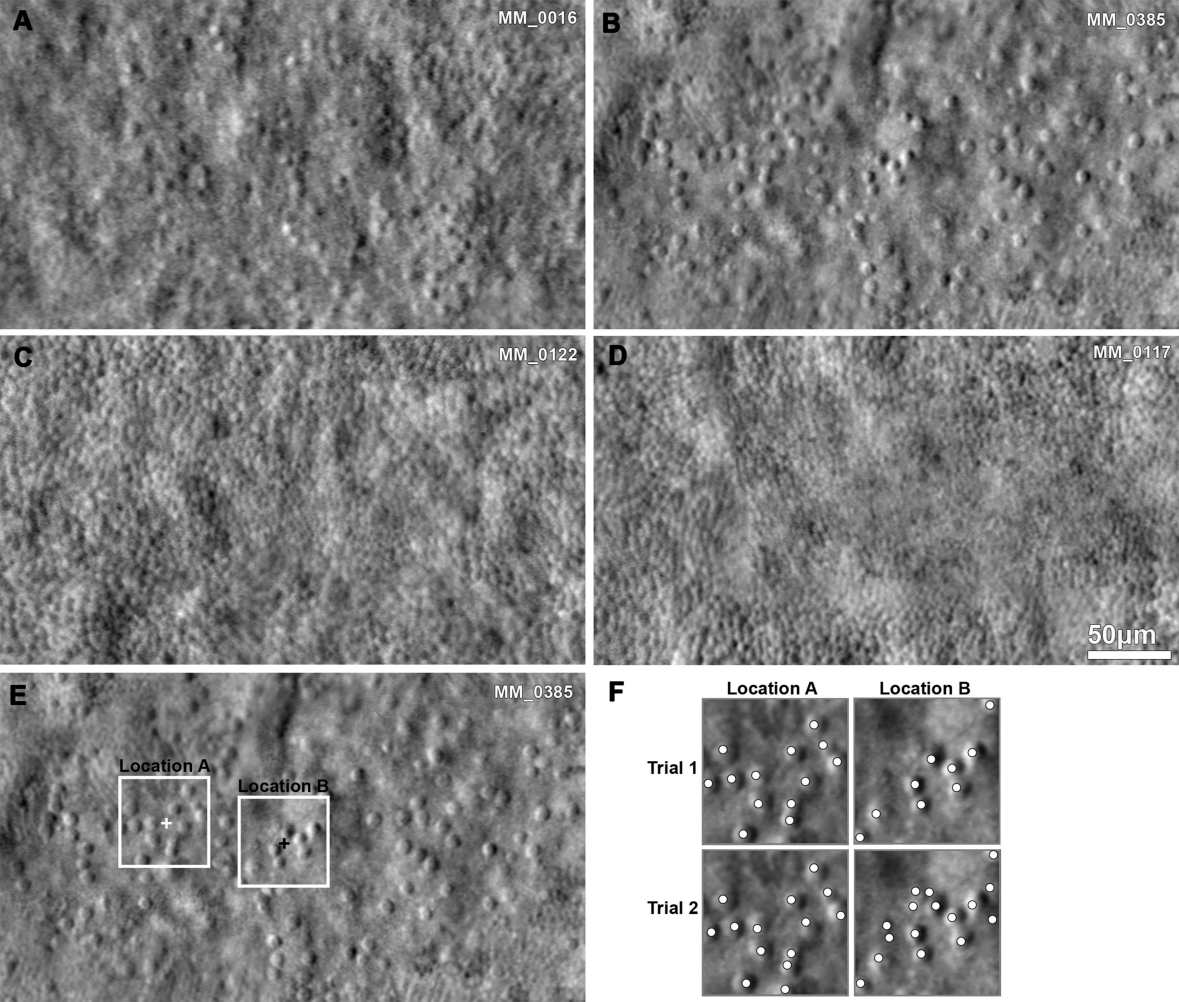
Examples of photoreceptor mosaics, reliability, and repeatability. (A–D) Split-detection images of the foveae identified having the highest disagreement in the intraobserver (*CNGA3*, MM_0016, MM_0385; *CNGB3*, MM_0117) or interobserver (*CNGA3*, MM_0016; *CNGB3*, MM_0122) Bland-Altman analysis. (A, B) Sparse mosaics of low foveal cone density. (C, D) Continuous mosaics of higher foveal cone density and limited resolution over the foveal center. (E) The *white squares* mark the 55-µm area region of interest (ROI) assessed for each of the two trials for observer 1 in the fovea (B). The *crosses* mark the two locations of maximum foveal cone density, with a displacement of 69 µm. (F) Higher magnification of the *squares* on (E), with the cone annotations for each trial (marked with *dots*). The reason for disagreement was attributed to the identification of ambiguous remnant cone-like structures during the second trial.

The foveae of all four subjects with highest disagreement are presented in Figures 3A–D. Two of the subjects had a sparse mosaic (MM_0016, MM_0385), and two had a continuous dense mosaic (MM_0122, MM_0117). For subject MM_0016, the image quality is postulated to be the reason for the intraobserver difference. Subject MM_0385 (Fig. 3B) had better image quality with a sparse mosaic and one of the lowest foveal cone density values in our cohort. For the last two subjects (MM_0122 and MM_0117), the combination of high density and low resolution over the foveal center is most likely the reason for the difference between trials.

### Displacement of Peak Cone Density and Cone Topography between Trials

The location of the maximum foveal cone density changed between each trial for each observer in all but one case. An example is shown in Figure 3E. The location of all maximum foveal cone density values in the 30 pairs of trials was recorded, and the distance between locations from trial 1 and trial 2 was calculated. For observer 1 and observer 2, the mean (SD; range) displacement of the maximum foveal cone density location was 38.51 (47.6; 2.97–248) µm and 44.71 (47.16; 0–203) µm, respectively. There was no correlation between the magnitude of displacement between trials and the cone density at that location (Spearman's correlation coefficient, *r* = –0.07, *P* = 0.713).

**Figure 4.**
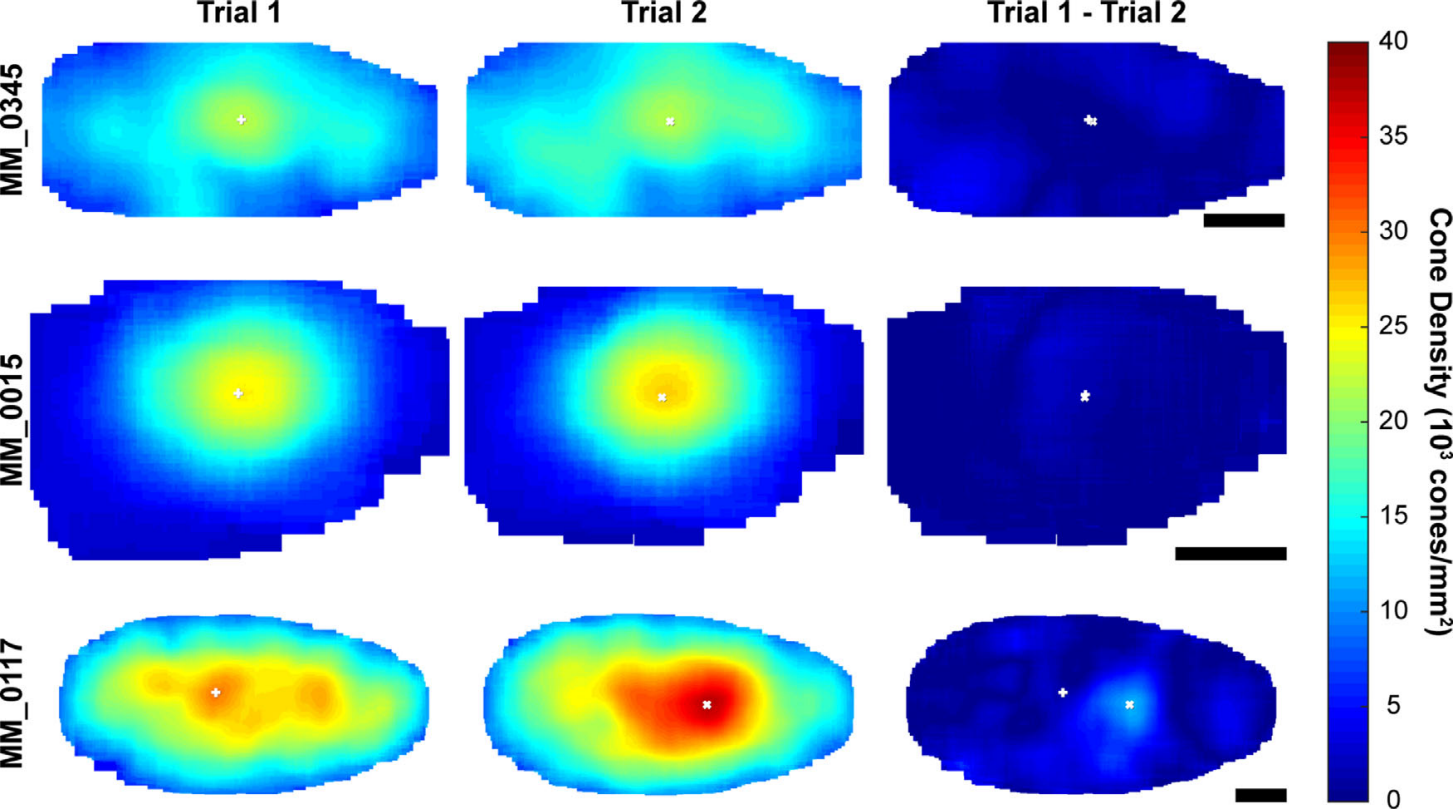
Foveal cone topography between trials. Foveal cone density maps for subject MM_0345 (*first row*) from observer 1, MM_0015 (*middle row*) from observer 2, and MM_0117 from observer 2 demonstrating examples of the smallest, mean, and largest absolute intraobserver difference in maximum foveal cone density, respectively. Cone density was calculated at every cone coordinate within the area counted. Difference maps show absolute difference between trial 1 and trial 2. *White crosses* (+ and x), location of maximum foveal cone density for trials 1 and 2. The displacement of maximum foveal cone density is 5.04 µm for MM_0345, 0.68 µm for MM_0015, and 115.83 µm for MM_0117. *Scale bar*: 100 µm.

We also examined the difference in cone topography between the trials for each observer. Three exemplar subjects (MM_0345, MM_0015, and MM_0117) with the smallest, mean, and largest absolute difference in maximum foveal cone density, respectively, are shown in Figure 4. The displacement of maximum foveal cone density is 5.04 µm for MM_0345, 0.68 µm for MM_0015, and 115.83 µm for MM_0117. In addition to differences in peak foveal cone density and its location, there were some regional differences in other areas of the ACHM fovea between trials 1 and 2.

### Comparison between *CNGA3* and *CNGB3* Achromatopsia

There was no statistically significant difference in maximum foveal cone density between the two genotypes when using the mean measurements for either observer 1 (two-sample *t*-test, *t* = 1.29, *P* = 0.208) or observer 2 (two-sample *t*-test, *t* = 1.26, *P* = 0.218).

## Discussion

In this study, we evaluated repeatability and reproducibility of foveal cone density measurements in *CNGA3*- and *CNGB3*-ACHM. It is the first report to investigate intraobserver repeatability and interobserver reproducibility of foveal cone density measurements for *CNGA3*-ACHM, as well as the first to evaluate interobserver reproducibility for *CNGB3*-ACHM.

Currently there are five phase I/II gene supplementation trials in total for *CNGB3*-ACHM (NCT03001310 and NCT02599922) and *CNGA3*-ACHM (NCT03758404, NCT02935517, and NCT02610582). Identification of robust structural measurements is crucial for patient stratification, as well as for safety and efficacy assessment. Outer nuclear layer (ONL) thickness, estimated from optical coherence tomography (OCT), is an *indirect* estimation of the residual photoreceptors nuclei, which may have a predictive value in the response to gene therapy in inherited retinal diseases.^37^ Recently, Mastey et al.^38^ reported a mean ONL thickness of 79.5 µm with a mean repeatability coefficient of 13.9 µm and excellent ICC, and proved symmetry among eyes in a large cohort of *CNGA3*- and *CNGB3*-associated ACHM (*n* = 76). AOSLO can *directly* visualize the residual photoreceptors, and cone density can be used as an additional measurement to ONL thickness. The values for ICC and the Bland-Altman analysis for foveal cone density indicate excellent intraobserver repeatability and interobserver reproducibility for both genotypes. However, there was a certain degree of bias in interobserver evaluation of both genotypes; hence, the clinical significance for cross-sectional assessment of patients remains uncertain. Our findings of excellent intraobserver repeatability for *CNGB3*-ACHM agree with previously reported ICC values for peak foveal cone density evaluated in split-detection images from a different cohort.^39^ The observed displacement of the locations of peak foveal cone density between trials is an interesting finding and of importance for any future AOSLO study in need of anatomic reference hallmarks in ACHM, including longitudinal natural history studies. The displacement in maximum foveal cone density location between trials may be related to the cone topography in ACHM (i.e., central photoreceptor disruption). The identification of the foveal center is crucial due to the anisotropy of the photoreceptor mosaic for defining meridians and for comparing locations across conditions and healthy controls. Further investigation of regularity metrics to better characterize continuous and sparse mosaics and the dislocation of maximal foveal cone density will be of value, as well as the use of an “independent anchor” (e.g., pit center in registered OCT images).

As previously discussed by Tanna et al.^29^ when assessing retinitis pigmentosa and Stargardt disease, reliability and repeatability of cone density estimates may be disease specific given the diversity of phenotypes across inherited retinal diseases. In our study, the two genotypes examined have similar reliability and repeatability, which is not surprising since *CNGA3*- and *CNGB3*-ACHM are clinically indistinguishable. However, the degree of repeatability and reliability may not extrapolate when evaluating cone metrics outside the fovea. In our study, both observers had significant experience in evaluating AOSLO images in ACHM, so these findings may not generalize to naive observers.^30^ Another limitation is the low acquisition rate (45/85, 53%) of successful (analyzable) AOSLO images in our ACHM cohort, compared to other retinal imaging modalities (e.g., OCT). Further investigation of predictive indicators for successful AOLSO acquisition and reliable analysis will be of value, including visual acuity, fixation stability, nystagmus quantification, and OCT appearance. While we performed our cone counting completely manually, there are automated algorithms for cone identification,^40^^–^^42^ which may facilitate more reliable cone density measurements in the ACHM fovea. However, these were trained on parafoveal images and do not perform well in tightly packed contiguous mosaics found in some ACHM foveae.

In conclusion, *CNGA3-* and *CNGB3*-ACHM can be successfully imaged with split-detection AOSLO, and the foveal cone mosaic can be evaluated with good intraobserver repeatability. The difference in measurements between observers emphasizes the need for longitudinal assessment to be completed by the same experienced observer. It also highlights the importance of pathology-specific training in cone counting and pathology-specific evaluation of automated methods for photoreceptor identification.^40^^,^^42^^,^^43^ Given its increasing use in clinical trials and natural history studies, there is a need for further studies to evaluate AOSLO metrics in different conditions.

## Supplementary Material

Supplement 1
