# Supplementary material for: Intraobserver Repeatability and Interobserver Reproducibility of Foveal Cone Density Measurements in CNGA3- and CNGB3-Associated Achromatopsia
Source: Transl Vis Sci Technol. 2020 Jun 26;9(7):37. doi: 10.1167/tvst.9.7.37 (PMC7414701; doi:10.1167/tvst.9.7.37)
Supplement: Supplement 1 [file tvst-9-7-37_s001.pdf]

Supplementary Table. Subject Demographics and Genetics

Subject ID	Sex	Age (years)	Variant 1		Variant 2	
			c.DNA Change	AA Change	c.DNA -Change	AA Change
**Gene: *CNGA3***						
JC_10069	M	18	847C>T	Arg283Trp	542A>G	Tyr181Cys
KS_10088	F	64	450-1G>A	fs	1157G>A	Met519Ile
KS_10337	F	17	940_942delATC	Ile214del	1114C>T	Pro372Ser
MM_0002	F	41	1443-1444insC	Ile482His fs*6/c	1706G>A	Arg569His
MM_0009	F	14	536T>A	Val179Asp	ND	ND
MM_0014	F	35	848G>A	Arg283Gln	667C>T	Arg223Trp
MM_0015	F	28	848G>A	Arg283Gln	667C>T	Arg223Trp
MM_0016	M	30	848G>A	Arg283Gln	667C>T	Arg223Trp
MM_0064	F	23	1694C>T	Thr565Met	661C>T	Arg221Ter
MM_0167	F	28	847C>T	Arg283Trp	1279C>T	Arg427Cys
MM_0171	M	22	1001C>T	Ser334Phe	1360A>T	Lys454Ter
MM_0239	M	37	848G>A	Arg283Gln	667C>T	Arg223Trp
MM_0385	M	37	1228C>G	Arg410Trp	1228C>G	Arg410Trp
MM_0386	F	22	1580T>G	Leu527Arg	1805G>A	Gly602Glu
MM_0398	F	14	811C>T	Pro271Ser	829C>T	Arg277Cys
**Gene: *CNGB3***						
MM_0005	M	27	1148delC	Thr383Ile fs * 13	607-608insT	Arg203Leu fs * 3
MM_0029	F	15	595delG	Glu199fs	1148delC	Thr383Ile fs * 13
MM_0040	M	38	1148delC	Thr383Ile fs * 13	1148delC	Thr383Ile fs * 13
MM_0116	M	24	595delG	Glu199fs	1148delC	Thr383Ile fs * 13
MM_0117	M	20	1148delC	Thr383Ile fs * 13	1148delC	Thr383Ile fs * 13
MM_0122	F	18	1148delC	Thr383Ile fs * 13	1148delC	Thr383Ile fs * 13
MM_0123	M	51	1148delC	Thr383Ile fs * 13	1148delC	Thr383Ile fs * 13
MM_0125	F	23	1148delC	Thr383Ile fs * 13	1148delC	Thr383Ile fs * 13
MM_0162	F	17	1148delC	Thr383Ile fs * 13	1148delC	Thr383Ile fs * 13
MM_0327	F	19	412delA	Arg138Glfs * 14	1432C>T	Arg478Ter
MM_0328	F	23	412delA	Arg138Glfs * 14	1432C>T	Arg478Ter
MM_0345	F	32	1148delC	Thr383Ile fs * 13	1148delC	Thr383Ile fs * 13
MM_0347	F	15	1148delC	Thr383Ile fs * 13	1148delC	Thr383Ile fs * 13
MM_0361	F	26	1148delC	Thr383Ile fs * 13	1148delC	Thr383Ile fs * 13
MM_0375	F	21	819_826del	Arg274Valfs* l3	1148delC	Thr383Ile fs * 13
